# Analysis of *Kudoa septempunctata* as a cause of foodborne illness and its associated differential diagnosis

**DOI:** 10.4178/epih.e2017037

**Published:** 2017-08-21

**Authors:** Ji-Hyuk Park

**Affiliations:** Department of Preventive Medicine, Dongguk University College of Medicine, Gyeongju, Korea

Dear Editor,

With great interest, I read the paper entitled, “Analysis of *Kudoa septempunctata* as a cause of foodborne illness and its associated differential diagnosis”, written by Lee [1], which was published in *Epidemiology and Health* in March 2017. The pathogenicity of *K. septempunctata* was advocated for in an article written by Kawai et al. [2] in Japan, but this proposal was argued against in 2 studies conducted by Ahn et al. [3] and Jang et al. [4] in South Korea (here-after Korea) using suckling mice. However, there are a few differences among these studies that should be noted.

First, the study by Kawai et al. [2] used *K. septempunctata* spores isolated from the cases of foodborne illness outbreaks, while the other studies [3,4] used *K. septempunctata* spores collected from commercial fish farms. Second, the genotypes of *K. septempunctata* can be classified as sequence type1 (ST1), ST2, and ST3. The ST1 and ST2 genotypes are mostly found in Japan, while the ST3 genotype is most commonly found in Korea [5]. No information was provided about the genotype of *K. septempunctata* in the study performed in Japan [2]. The 2 studies performed in Korea [3,4] assessed the pathogenicity of the ST3 genotype. Thus, these findings do not demonstrate the lack of pathogenicity of the ST1 and ST2 genotypes.

According to the study by Lee [1] in Korea, 11 foodborne outbreaks in 2015 provide support for the possibility of *K. septempunctata* pathogenicity. However, there are limitations to this argument because those outbreak investigations were case series studies, not case-control studies or retrospective cohort studies. In Japan, among 24 foodborne outbreaks with unknown causes, 4 outbreaks were significantly associated with the consumption of olive flounder (*Paralichthys olivaceus*). Among those 4 outbreaks, *K. septempunctata* was isolated from at least 1 foodborne outbreak [2].

In Korea, *K. septempunctata* has not been included as a causative agent of foodborne outbreaks in the 2016 guidelines for water and foodborne diseases prevention and control [6]. However, *K. septempunctata* was included as a causative agent of food poisoning in the 2017 food safety administrative guidelines [7]. To obtain more epidemiological evidence of pathogenicity, further efforts are needed to perform a case-control study or cohort study of foodborne outbreaks relating to the consumption of olive flounder. Moreover, more stringent traceback investigations are needed to determine the production and distribution chain of olive flounder.
